# Complete genome sequence of a natural compounds producer, *Streptomyces violaceus* S21

**DOI:** 10.1016/j.gdata.2017.04.002

**Published:** 2017-04-28

**Authors:** Jiafang Fu, Chuanqing Zhong, Zhilong Zhao, Gongli Zong, Guangxiang Cao

**Affiliations:** a Shandong Medicinal Biotechnology Centre, Shandong Academy of Medical Sciences, Jinan, China; b Shandong Jianzhu University, Jinan, China; c College of Drug, Linyi University, Linyi, China

**Keywords:** *Streptomyces violaceus*, Complete genome sequence, Natural compounds, Anthracycline antibiotics

## Abstract

The complete genome sequence of *Streptomyces violaceus* strain S21, a valuable natural compounds producer isolated from the forest soil, is firstly presented here. The genome comprised 7.91M bp, with a G + C content of 72.65%. A range of genes involved in pathways of secondary product biosynthesis were predicted. The genome sequence is available at DDBJ/EMBL/Genbank under the accession number CP020570. This genome is annotated with 6856 predicted genes identifying the natural product biosynthetic gene clusters in *S. violaceus*.

**Table:** 

Specifications	
Organism/cell line/tissue	*Streptomyces violaceus*
Sequencer or array type	Illumina Hiseq4000, PacBio RS II
Data format	Raw and processed
Experimental factors	Microbial strains
Experimental features	Complete genome sequence of *Streptomyces violaceus* strain S21, assembly and annotation
Consent	N/A
Sample source location	The Seabed sludge (Shandong Province, China) (36°67′5.7″ N, 122°98′28.0″ E).

## 1 Direct link to deposited data

The complete genome sequences can be found at the site https://www.ncbi.nlm.nih.gov/nuccore/CP020570.

The raw data can be found at the site http://dx.doi.org/10.17632/66hfym3csx.1.

## 2 Introduction

Natural products from actinomycetes have been the major sources for clinical antibiotics, along with numerous other useful compounds including antineoplastic, antiparasitic, insecticidal and phytocidal drugs. *Streptomyces violaceus* is one of the great potential producers of natural compounds. *S. violaceus* was first isolated and classified in the 1960s [1]. Following its discovery, different kinds of anthracycline antibiotics for cancer treatment were isolated from *S. violaceus*, but side effects like cardiotoxicity have limited their clinical use [2], [3], [4], [5]. Development of new anthracyclines with less cardiotoxicity and improved therapeutic efficacy is required. In addition, amylase inhibitors, extracellular polysaccharide and thrombolytic actinoprotease were also isolated from *S. violaceus* [6], [7], [8]. *S. violaceus* was also used to develop new useful natural products recently [9], [10]. Until now, only one draft genome of *S. violaceus* NRRL B-2867 has been deposited in Genbank [9], [10]. To further understand this potential producer of many natural compounds, we present the first complete genome sequence of *S. violaceus* S21 and its features.

## 3 Experimental design, materials and methods

Strain S21 was isolated from the Seabed sludge in Shandong, China. Strain S21 is a valuable producer of many natural compounds, including anthracycline antibiotics, amylase inhibitors and extracellular polysaccharide. Analysis of the genome of strain S21 was carried out in order to reveal the biosynthetic gene clusters of natural compounds.

*S. violaceus* S21 was cultured in Tryptic Soy Broth (OXOID, UK) medium to obtain mycelium, then Genomic DNA was extracted using Genomic DNA Purification Kit (Promega, USA). Both the PE300 DNA library and 10-kb DNA library were constructed, after the quality of DNA sample was analyzed using a NanoDrop 2000 Spectrophotometer (Thermo Scientific, USA). DNA sequencing was performed using an Illumina Hiseq4000 platform and a PacBio RS II platform at Beijing Genomics Institute (Shenzhen, China). The cleaned reads were *de novo* assembled with SPAdes [11], then polished with SSPACEStandard and GapFiller to get scaffolds [12], [13]. The genome was annotated using the Prokaryotic Genome Annotation Pipeline (PGAP) version 3.2 software on NCBI. Additional gene prediction was performed by the RASTtk server [14]. SEED viewer was used for assignment of the predicted genes to functional categories [15].

## 4 Data description

**Fig. 1:**
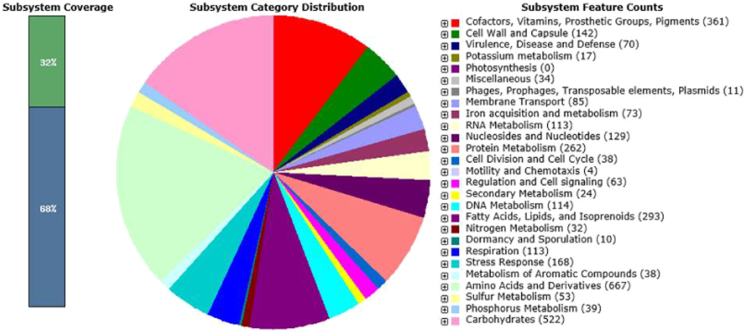
Subsystems of *S. violaceus* S21 based on SEED database.

After quality control, about 1.50 Gb of data was obtained from the Illumina Hiseq platform, and about 0.81 Gb of data was obtained from the PacBio RS II platform. A total of 7,916,045 bp genome sequence with an average GC content of 72.65% was assembled. The genome was predicted to contain 6856 genes, including 6571 coding sequences, 65 tRNAs, 18 rRNAs (5S, 16S, and 23S), 3 ncRNAs, and 199 pseudo genes. Most of the annotated genes determined amino acids and derivative synthesis (667), carbohydrate metabolism (409), cofactor, vitamin, prosthetic group and pigment formation (357), protein metabolism (349), and fatty acid, lipid and isoprenoid (149) (Fig. 1).

About 79 gene clusters involved in the pathways for the secondary products biosynthesis were predicted in the genome of Strain S21 using antiSMASH [16]. Further studies of the genes involved in the biosynthesis of anthracycline antibiotics, amylase inhibitors and extracellular polysaccharide are necessary.

## 5 Nucleotide sequence accession numbers

The nucleotide sequence of the *S. violaceus* S21 genome has been deposited in Genbank under the accession number CP020570.

## Conflict of interest

The authors declare that there is no conflict of interests on the work published in this paper.

## References

[1] Munoz E., Elorza M.V., Villanueva J.R. (1964). Classification of a microorganism with lytic action as a type of *Streptomyces violaceus*. Microbiol. Esp..

[2] Johdo O., Ishikura T., Yoshimoto A., Takeuchi T. (1991). Anthracycline metabolites from *Streptomyces violaceus* A262. I. Isolation of antibiotic-blocked mutants from *Streptomyces violaceus* A262. J. Antibiot..

[3] Johdo O., Yoshioka T., Takeuchi T., Yoshimoto A. (1997). Isolation of new anthracyclines 10-O-rhodosaminyl beta-rhodomycinone and beta-isorhodomycinone from mild-acid treated culture of obelmycin-producing *Streptomyces violaceus*. J. Antibiot..

[4] Miyamoto Y., Johdo O., Nagamatsu Y., Yoshimoto A. (2002). Cloning and characterization of a glycosyltransferase gene involved in the biosynthesis of anthracycline antibiotic beta-rhodomycin from *Streptomyces violaceus*. FEMS Microbiol. Lett..

[5] Miyamoto Y., Ohta S., Johdo O., Nagamatsu Y., Yoshimoto A. (2000). Production of a new hybrid anthracycline 4-O-methylepelmycin by heterologous expression of dnrK in epelmycin-producing *Streptomyces violaceus*. J. Antibiot..

[6] Manivasagan P., Sivasankar P., Venkatesan J., Senthilkumar K., Sivakumar K., Kim S.K. (2013). Production and characterization of an extracellular polysaccharide from *Streptomyces violaceus* MM72. Int. J. Biol. Macromol..

[7] Mohanasrinivasan V., Subathra C.D., Yogesh S., Govindaraj A., Jemimah S.N. (2016). In vitro thrombolytic potential of actinoprotease from marine Streptomyces violaceus VITYGM. Cardiovasc. Hematol. Agents Med. Chem..

[8] Sharova N. (2015). Amylase inhibitors from *Streptomyces lucensis* VKPM Ac-1743 and *Streptomyces violaceus* VKPM Ac-1734. Prikl. Biokhim. Mikrobiol..

[9] Doroghazi J.R., Albright J.C., Goering A.W., Ju K.S., Haines R.R., Tchalukov K.A., Labeda D.P., Kelleher N.L., Metcalf W.W. (2014). A roadmap for natural product discovery based on large-scale genomics and metabolomics. Nat. Chem. Biol..

[10] Ju K.S., Gao J., Doroghazi J.R., Wang K.K., Thibodeaux C.J., Li S., Metzger E., Fudala J., Su J., Zhang J.K., Lee J., Cioni J.P., Evans B.S., Hirota R., Labeda D.P., van der Donk W.A., Metcalf W.W. (2015). Discovery of phosphonic acid natural products by mining the genomes of 10,000 actinomycetes. Proc. Natl. Acad. Sci. U. S. A..

[11] Bankevich A., Nurk S., Antipov D., Gurevich A.A., Dvorkin M., Kulikov A.S., Lesin V.M., Nikolenko S.I., Pham S., Prjibelski A.D., Pyshkin A.V., Sirotkin A.V., Vyahhi N., Tesler G., Alekseyev M.A., Pevzner P.A. (2012). SPAdes: a new genome assembly algorithm and its applications to single-cell sequencing. J. Comput. Biol..

[12] Boetzer M., Henkel C.V., Jansen H.J., Butler D., Pirovano W. (2011). Scaffolding pre-assembled contigs using SSPACE. Bioinformatics.

[13] Boetzer M., Pirovano W. (2012). Toward almost closed genomes with GapFiller. Genome Biol..

[14] Brettin T., Davis J.J., Disz T., Edwards R.A., Gerdes S., Olsen G.J., Olson R., Overbeek R., Parrello B., Pusch G.D., Shukla M., Thomason J.A., Stevens R., Vonstein V., Wattam A.R., Xia F. (2015). RASTtk: a modular and extensible implementation of the RAST algorithm for building custom annotation pipelines and annotating batches of genomes. Sci. Rep..

[15] Overbeek R., Olson R., Pusch G.D., Olsen G.J., Davis J.J., Disz T., Edwards R.A., Gerdes S., Parrello B., Shukla M., Vonstein V., Wattam A.R., Xia F., Stevens R. (2014). The SEED and the Rapid Annotation of microbial genomes using Subsystems Technology (RAST). Nucleic Acids Res..

[16] Weber T., Blin K., Duddela S., Krug D., Kim H.U., Bruccoleri R., Lee S.Y., Fischbach M.A., Muller R., Wohlleben W., Breitling R., Takano E., Medema M.H. (2015). antiSMASH 3.0-a comprehensive resource for the genome mining of biosynthetic gene clusters. Nucleic Acids Res..

